# Not an infection: Endogenous circoviral elements underlie BFDV detections in Old World vultures

**DOI:** 10.1371/journal.pone.0351507

**Published:** 2026-06-15

**Authors:** Iñigo Palacios-Martínez, Francisco Morinha, Guillermo Blanco

**Affiliations:** 1 Department of Evolutionary Ecology, Museo Nacional de Ciencias Naturales, CSIC, José Gutiérrez Abascal 2, Madrid, Spain; 2 Morinha Lab-Laboratory of Biodiversity and Molecular Genetics, Vila Real, Portugal; Institute of Biochemistry and Biophysics Polish Academy of Sciences: Instytut Biochemii i Biofizyki Polskiej Akademii Nauk, POLAND

## Abstract

Circoviruses (family Circoviridae) are small, single-stranded DNA viruses known to infect a broad range of bird species. The Beak and Feather Disease Virus (BFDV), typically associated with parrots, has occasionally been reported in unrelated avian taxa, raising questions about host range and potential spillover. We investigated BFDV-like sequences in three sympatric vultures breeding in Spain (Egyptian vulture *Neophron percnopterus*, cinereous vulture *Aegypius monachus*, and griffon vulture *Gyps fulvus*). PCR screening revealed low and inconsistent prevalence in Egyptian vultures (7.4%, n = 163) and cinereous vultures (2.9%, n = 47), and absent in griffon vultures (n = 35). Phylogenetic analyses identified nine highly divergent haplotypes spanning three distinct clades, with no structure by species, geography, or year, and no evidence of nest- or territory-level transmission. These patterns contradict expectations for an actively circulating virus and instead suggest that the sequences represent endogenous circoviral elements (ECV) integrated into the host genome. The absence of clinical signs, except in one inbred Egyptian vulture nestling (offspring of a brother-sister mating), indicates these elements are non-pathogenic. Our findings demonstrate that BFDV detections in non-psittacine birds often reflect genomic remnants rather than active infections. This reinterpretation underscores the need to combine molecular surveillance and evolutionary genomics to distinguish actual infections from viral relics.

## Introduction

Beak and feather disease virus (BFDV), a member of the family Circoviridae with single-stranded DNA (ssDNA) genomes and a high mutation rate [1,2], comprises genetic sequences frequently associated with psittacine beak and feather disease (PBFD). This is a chronic and often fatal pathology of Psittaciformes characterized by feather loss and beak deformities [3] that contributes significantly to morbidity and mortality in both wild and captive populations, including threatened species [4,5]. Although detected in multiple psittacine species, disease severity varies considerably both across and within species [1]. BFDV sequences have been detected in some clinically normal individuals, whereas others exhibiting signs consistent with PBFD may test negative for these sequences [6–8], indicating that clinical manifestations can arise from other metabolic, developmental, pathogenic, or toxic processes, and that the presence of BFDV sequences does not necessarily imply disease expression. The high prevalence and genetic diversity in wild psittacine populations throughout Australia and neighbouring regions [9–11] strongly suggests that BFDV originated there [12,13].

Reports of BFDV sequences in non-psittacine birds are rare [14] and may reflect incidental exposure in areas shared with native psittacines [2]. The global presence of BFDV has been largely associated with the international trade of captive birds [15,16], which has been hypothesized to pose a potential, yet untested, risk to native avifauna from introduced species [17,18]. Parrots are known to perform long-distance movements, and overlapping ranges with other avian species may lead to the detection of BFDV sequences across widespread regions, including areas where psittacines are non-native invasive species, resembling patterns observed for pathogens with well-established epidemiology [19,20]. However, the molecular and ecological mechanisms governing the potential circulation of BFDV remain unresolved. Several studies have shown that members of the Circoviridae family have integrated fragments of their genomes into the DNA of various vertebrate lineages, forming endogenous circoviral elements (CVe) [21,22]. These CVe are non-infectious “viral fossils” that persist in host genomes over millions of years, providing insights into the deep evolutionary history of circoviruses [22,23]. This raises the possibility that some PCR-based detections of BFDV in birds might correspond to integrated, non-replicating ECV rather than active viral infections.

Very little is known about the presence of BFDV in Europe, where no native parrots occur, but several introduced species maintain growing populations [24]. A study conducted in Seville, southern Spain, detected the same BFDV sequence in two introduced parakeet species, the monk parakeet (*Myiopsitta monachus*) and the neck-ringed parakeet (*Alexandrinus krameri*), with relatively high prevalence but without any clinical signs of PBFD [18]. In contrast, sampling of a variety of native bird species sharing the area with these introduced parrots revealed neither clinical signs nor the presence of these or other BFDV sequences [25]. Additionally, a study in central Spain reported the presence of BFDV in nestlings of the Egyptian vulture (*Neophron percnopterus*) in a region devoid of invasive psittacine species [26].

In this study, we assessed the prevalence and phylogenetic placement of BFDV variants found in the Egyptian vulture, Griffon vulture (*Gyps fulvus*), and Cinereous vulture (*Aegypius monachus*) breeding in sympatry in Europe, whose migratory movements may bring it into variable contact with wild psittacine populations in African wintering grounds [27–29]. We specifically tested whether the BFDV sequences detected in vultures may represent an infectious agent capable of circulating within and between species. Alternatively, these sequences may represent non-infectious relicts, a possibility that would have profound implications for understanding BFDV evolution, host specificity, and the interpretation of molecular surveillance data. From the hypothesis that BFDV sequences detected in vultures represent an infectious agent capable of circulating within and between species, either among related individuals (e.g., parents and offspring, siblings) or among unrelated individuals through nest reuse or contact at shared feeding sites (exogenous hypothesis), we derived three predictions: (1) BFDV prevalence will be low across vulture species, reflecting their limited interaction with psittacines, but higher in Egyptian vultures than in griffon vultures, and lower than in cinereous vultures, consistent with their respective exposure potential during African overwintering; (2) sibling Egyptian vultures and nestlings from the same nests in different years, will show higher prevalence and a greater likelihood of sharing viral variants than unrelated individuals, suggesting potential direct transmission within nests; (3) viral variants detected in vultures will be genetically closer to African BFDV sequences than to those from other regions, supporting exposure in African wintering grounds rather than exposure from feral and captive psittacines in the European breeding grounds. The alternative hypothesis of integrated ECV (endogenous hypothesis) predicts that (1) BFDV-like sequences detected will show high sequence conservation across individuals and sampling years, consistent with a stable genomic insertion; (2) sibling nestlings or individuals from the same nests in different years may differ in their BFDV detection status and variants, reflecting Mendelian inheritance of a genomic fragment; and (3) phylogenetic analyses of the detected sequences are expected to show high similarity to genomic regions of bird species from different continents, even from those not known to harbour BFDV.

## Materials and methods

### Study species and study area‌‌

The Egyptian vulture is a small (~2 kg) obligate scavenger inhabiting open, arid, and mountainous landscapes across southern Europe, Africa, and Asia [30,31]. This species nests on rocky cliffs, where it defends territories during the breeding season. It forages both solitarily and socially, depending on food availability and season [32]. Its diet consists mainly of carrion, including wild animals such as ungulates, lagomorphs, birds, and reptiles, as well as domestic livestock remains [33,34].

Egyptian vultures are monogamous, and highly philopatric, with a slow life history strategy characterized by delayed reproduction (on average, at 7 years of age), low fecundity, and high longevity (up to 24 years in the wild) [35]. The species has a low reproductive output, with a clutch size of two eggs and 0–2 fledglings per breeding attempt. Globally, the Egyptian vulture is listed as Endangered due to significant population declines across much of its range, with the majority of European populations concentrated in Spain, where there has been a marked decline in breeding numbers in recent decades [36].

Egyptian vultures from Spain migrate through the Sahara-Sahel corridor during the non-breeding period [29]. Their wintering grounds overlap with several species of African psittacines [37]. In addition, although Egyptian vultures do not naturally coexist with native Psittacidae species in their breeding range on Europe, their habitat requirements and the predominantly urban distribution of feral parrot populations [24] make spatial overlap highly unlikely, except occasionally in some humanized areas.

**Fig 1:**
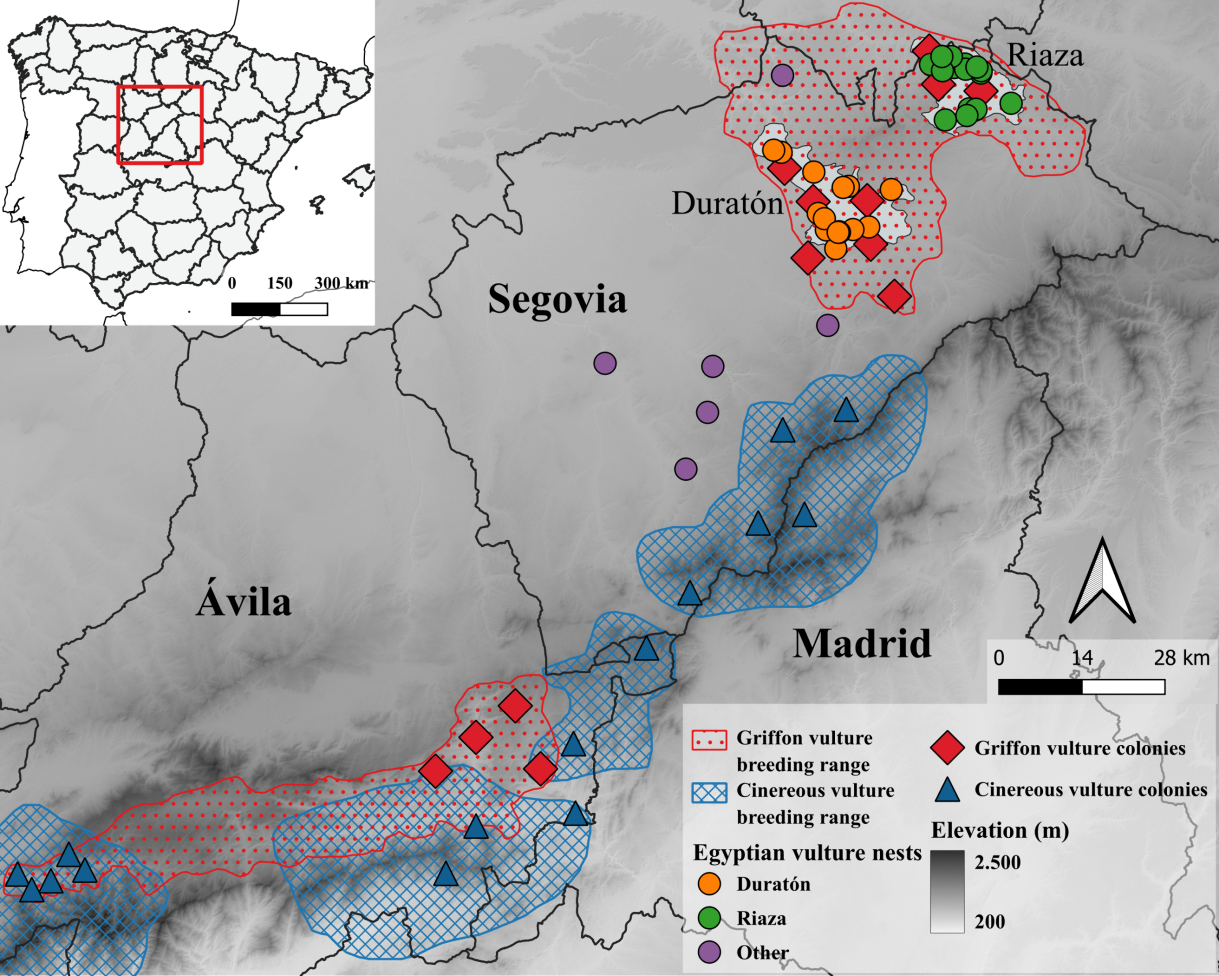
Study area of Old World vultures in Ávila, Madrid, and Segovia provinces, Central Spain. Egyptian vulture (*Neophron percnopterus*) nests are indicated by dots, color-coded by location: red for nests in Hoces del Río Riaza Natural Park, orange for Hoces del Río Duratón Natural Park, and purple for nests in other areas, out of the natural parks. The cinereous vulture (*Aegypius monachus*) breeding area (blue shape) and colonies (blue triangles) are marked along the Central Mountain Range. The griffon vulture (*Gyps fulvus*) breeding range (red shape) and studied colonies (red diamonds) are shown, with most individuals concentrated within the two Natural Parks. Both Natural Parks are labelled and indicated in grey. In the upper left corner, an inset map of the Iberian Peninsula shows the location of the study area. Background topography is based on a 30 m resolution digital elevation model from NASA Earth Observatory (http://earthobservatory.nasa.gov/). The regional boundaries of Spain were mapped using cartographic layers from the freely available database of the Instituto Geográfico Nacional (IGN; https://www.ign.es/web/ign/portal). Country borders were obtained from Natural Earth (https://www.naturalearthdata.com/).

This study was conducted in the provinces of Ávila, Madrid and Segovia, central Spain (Fi. 1), which support a small and declining population of Egyptian vultures comprising approximately 27 breeding pairs [26]. The majority of these pairs nest within two major protected areas: the Hoces del Río Duratón and Hoces del Río Riaza Natural Parks. Several isolated pairs occupy smaller, satellite cliff sites surrounding these protected areas (Fig 1). The region is also home to other scavenger species, including the cinereous vulture with an estimated population about 580 pairs distributed across the Central Mountain Range [38], and the griffon with about 2,000 pairs, mostly concentrated within the Duratón and Riaza Natural Parks [39]. No feral parrot populations have been reported in the study area.

### Fieldwork and sampling procedures

Over the past three decades, a comprehensive long-term monitoring program has been conducted in the study area. This program focused on systematically locating Egyptian vulture territories, ringing nestlings, and identifying ringed breeding individuals within these territories [35,40]. Nest access was carefully planned, with climbers reaching the nests when chicks were approximately 50 days old.

Egyptian vulture clutches typically consist of two eggs, but broods may result in one or two chicks, as the younger sibling often dies [41]. Therefore, sampled chicks could either be a single chick (brood size = 1) or two siblings (brood size = 2) from the same nest. Hatching order was assessed in double broods according to differences in nestling body size (mainly weight, and wing and tail length) and plumage development. Body condition was estimated as the residuals of a standardized major axis regression of log^10^ of weight against log^10^ of structural body size (tarsus length; type-2 standardized major axis regression -SMA-) [42]. Laying date was defined as the date on which the first egg of the clutch was laid (in days, from 1st March). This date was inferred by back-calculation based on nestling age estimated from wing feather growth [43].

During the study period (2001–2025), nests of cinereous and griffon vultures were also accessed within the same breeding areas to ring the nestlings with uniquely identifiable metal and plastic alphanumeric rings for long-distance identification, and to take biometrical measures and biological samples for research. A total of 688 nestlings from these three vulture species were sampled.

Each nestling was examined for visible signs of PBFD-compatible symptoms, and blood samples were collected from the brachial vein of 245 chicks across the three species for molecular sexing [44], and molecular BFDV screening.

### Ethics statement

This study complied with ethical standards established under Spanish legislation (Royal Decree 1205/2005) concerning the protection of animals used in scientific research. Fieldwork was conducted under permits issued by the Spanish Bird Ringing Centre (Permit No.: 530115) and the Regional Government of Castilla y León (Expte: EP/CyL/298/2016). The names of ravines and cliffs, and the precise locations of the nests of the studied scavenger birds are kept confidential to avoid disturbance during the breeding season.

### DNA extraction and BFDV screening

Samples were analyzed for the presence of avian circovirus using molecular techniques. Genomic DNA was extracted from blood samples using the Quick-DNA Miniprep Kit (Zymo Research, Irvine, CA, USA), following an optimized protocol described by Morinha et al. [18]. DNA concentration and quality were assessed using a Qubit 3.0 fluorometer (Thermo Fisher Scientific, Sunnyvale, CA, USA) and by agarose gel electrophoresis. A pre-PCR amplification with the molecular sexing primers 2550F/2718R [44] were performed to exclude the presence of potential PCR inhibitors. The screening of avian circovirus was carried out using four previously described molecular markers to include a wide range or circovirus strains detected in various bird orders (S1 Table). PCR was performed in a reaction mixture of 10 μl containing 5 μl of 2x MyTaq HS Mix (Bioline), 2.5 µM of each primer and ~20 ng of template DNA. The amplification protocol was composed of the following steps: 95°C for 5 min followed by 40 cycles of 95°C for 30 s, annealing temperature optimized for each marker (S1 Table) for 1 min, 72°C for 30 s, and a final extension at 60°C for 10 min. Positive, negative, and non-template controls were always included to rule out contamination issues. BFDV positive samples (all samples with an accurate and specific PCR amplification of the expected size fragment) were bi-directionally sequenced by Sanger sequencing to analyse its diversity and relationship with other circovirus strains.

### Statistics and phylogenetic analyses

BFDV prevalence was compared among species and across years using contingency tables and Fisher’s exact test (fisher.test function, *stats* package v.4.3.1) [45]. Generalized linear mixed models (GLMMs, binomial error distribution) were used to examine the effects of sex, brood size, hatching order, nest location, body condition, and laying date on BFDV occurrence in nestling Egyptian vultures with glmmTMB function (*glmmTMB* package v3.6) [46], and model selection and averaging (ΔAICc < 2) were performed using the model.sel and model.avg functions from the *MuMIn* package v.1.47.5 [47]. All models included the year and the nest ID as a random factor.

Differences in genetic distances between BFDV sequences were evaluated using the non-parametric Kruskal–Wallis test (kruskal.test function), and pairwise comparisons of genetic distances among and within years were further tested with Wilcoxon rank-sum tests (wilcox.test function), implemented in R v.4.3.1 [45].

The BFDV sequences obtained from vulture hosts were initially analyzed using phylogenetic methods to determine their evolutionary relationships. These sequences were then placed within the broader context of viral diversity by incorporating wild host sequences available from the GenBank database [48]. Phylogenetic reconstructions were performed using Bayesian Inference (BI), both for sequences derived solely from vulture hosts, and in combination with sequences from wild hosts. These analyses were implemented in MrBayes v.3.2.3 [49]. Analyses started with a randomly generated tree, and four Markov Chain Monte Carlo (MCMC) chains were run for 1,000,000 generations, with trees sampled every 1,000 generations. The first 25% of trees were discarded as burn-in, and a consensus tree was constructed from the remaining trees. Substitution models were specified using the lset nst = mixed, rates = invgamma option to account for variable substitution types and among-site rate variation. The final consensus trees were visualized and edited using FigTree v1.4.4 [50]. Bayesian posterior probabilities (BPPs) were used to assess nodal support.

To complement and extend the phylogenetic framework, sequence similarity searches were performed using the BLAST algorithm to explore the taxonomic origin and potential integration of BFDV-like sequences. First, viral sequences were compared against GenBank using BLASTn (≥95% sequence identity) to identify closely related variants and assess whether vulture-derived BFDV sequences clustered with those from captive or wild hosts. To further test the hypothesis of endogenous origin, a BLASTn search was conducted against the GenBank whole-genome shotgun (WGS) contig database, restricted to avian taxa (taxid: 8782; ≥ 20% query cover; BLASTg1). Finally, a representative full-length BFDV genome (HM748919) from *Poicephalus robustus* was used in an additional BLAST search (BLASTg2) to provide a comprehensive overview of misassigned BFDV-like sequences across avian genomes.

## Results

### Prevalence of BFDV across species and years

BFDV prevalence was 7.4% in Egyptian vultures (95% CI: 3.9–12.5%, n = 163) and 2.9% in cinereous vultures (95% CI: 0.1–14.9%, n = 35). Prevalence did not differ significantly between the two species (Fisher’s exact test, p = 0.10). In contrast, all griffon vulture samples tested negative (n = 47). Positive samples in *N. percnopterus* (Np) were detected in 2005 (16.7%; 2/12), 2006 (10.5%; 2/19), 2008 (20.0%; 1/5) 2009 (14.3%; 1/7), 2010 (33.3%; 3/9), 2013 (9.1%; 1/11), 2016 (6.3%; 1/16), and 2018 (7.1%; 1/14), while *A. monachus* (Am) showed positives only in 2010 (16.7%; 1/6). In the remaining years 2004 (nNp = 8, nAm = 3), 2007 (nNp = 4), 2011 (nNp = 11), 2014 (nNp = 10, nAm = 2), 2015 (nNp = 13), 2017 (nNp = 11, nAm = 1), 2019 (nNp = 3), 2020 (nNp = 13, nAm = 10), BFDV was not detected (S1 Table). Overall, BFDV prevalence did not differ significantly among sampling years pooling vulture species (Fisher’s exact test, p = 0.09).

### Factors influencing BFDV in Egyptian vulture nestlings

BFDV was detected in 22.2% of Egyptian Vulture nests (8 out of 36). Within these eight positive nests, 12 individuals tested positive and 60 tested negative (prevalence of 16.7% among susceptible individuals within positive nests). No siblings from the same nest and breeding season tested positive concurrently. No significant effects of nest of origin, year, sex, brood size (1–2 nestlings), hatching order (single, first, or second), nest location (Riaza, Duratón, and other areas), laying date, or body condition were detected on BFDV presence (GLMM, binomial error; S2 Table). In addition, the total number of nestlings per nest sampled across the years did not significantly influence the likelihood of being positive (GLMM, binomial error: Intercept = 0.02, p = 0.71).

### Apparent symptomatology of PBFD in vulture nestlings

Only one Egyptian vulture nestling out of 196 sampled individuals (0.5%; individual 9MC, 2018) exhibited visible clinical signs consistent with PBFD. This individual was the only among the 12 that tested positive for BFDV, whereas no clinical signs were observed in any of the BFDV-negative birds. Its full sibling (9MF), sampled in 2020, appeared healthy and tested negative. No external plumage or beak abnormalities indicative of PBFD were observed in cinereous vultures (n = 141) or griffon vultures (n = 351) nestlings.

### Genetic diversity and variation of BFDV sequences

Successful amplification of BFDV fragments was achieved exclusively using the primer pair from Amery-Gale et al. [14], yielding viral amplicons of 491 bp and 494 bp (GenBank accessions: PX597144 to PX597156; S3 Table). All attempts to amplify other circoviral sequences and the complete circovirus genome failed. Considerable nucleotide variation was observed among the nine different BFDV haplotypes found in Egyptian and cinereous vultures, with 0–67 substitutions (range = 0–13.6%). The mean pairwise sequence identity was 91.4 ± 4.2% (S4 Table). Genetic distances between BFDV haplotypes did not differ significantly across years (Kruskal-Wallis χ² = 2.36, df = 2, p = 0.31), nor among nests of Egyptian vultures (χ² = 3.0, df = 3, p = 0.39). Likewise, no significant differences were found between inter-annual and intra-annual distances (Wilcoxon W = 255, p = 0.69), nor between within-nest and among-nest comparisons (Wilcoxon W = 91.5, p = 0.39). Examples of high similarity between sequences from different years and nests were chicks 3C7 and 32M (from 2013 and 2018, in different nests) with 100% identity. In contrast, more divergent pairs included sequences from the same year (e.g., 1V9–246 in 2005, 87.2%) and the same nest but in different years (e.g., 246 in 2005–27L in 2009, in the same nest, 87.4%; S4 Table).

### Phylogeographic and phylogenetic relationships of BFDV in vultures

**Fig 2:**
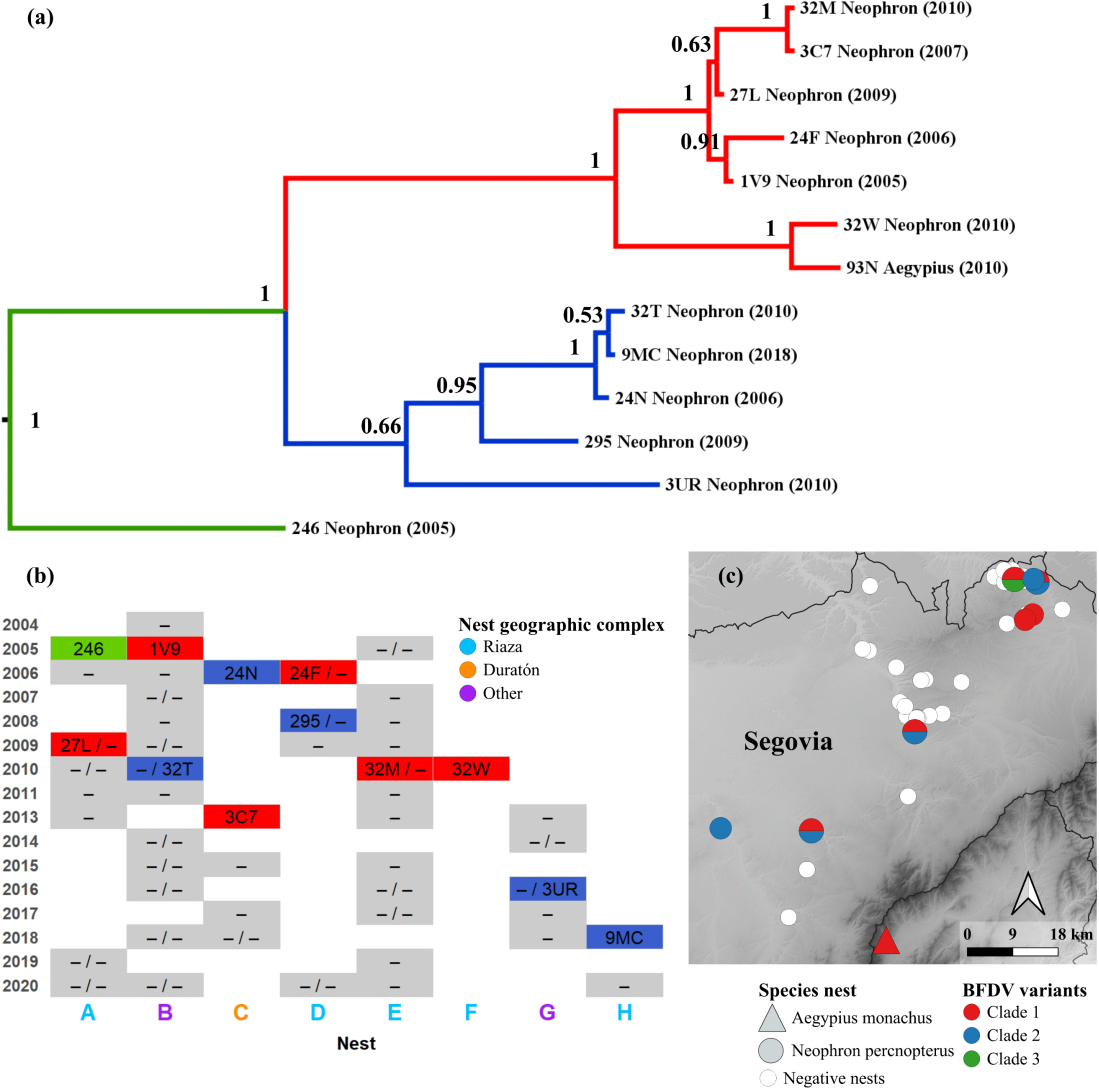
Phylogenetic and spatial and nest patterns of BFDV diversity in Old World vultures. **(a)** Phylogenetic tree of BFDV sequences obtained from vultures. Each sequence is labelled with the corresponding alphanumeric ring ID and the sampling year (in brackets). Numbers at branch nodes correspond to BPPs (Bayesian Posterior Probabilities). Genetic clades are color-coded as in panels (a) and **(b)**: red for Clade 1, blue for Clade 2, and green for Clade 3. **(b)** Schematic representation of BFDV-positive nests in Egyptian vultures (*Neophron percnopterus*). Each nest shows the status of individual nestlings: positive individuals are labelled with their alphanumeric ring ID, while negative individuals are indicated with a “(-).” In nests with two chicks, individuals are separated by “/”; the first hatching nestling is shown on the left, and the second on the right. Nests containing only negative individuals are shaded in grey, whereas nests with at least one BFDV-positive chick are color-coded according to the viral genetic clade: red for Clade 1, blue for Clade 2, and green for Clade 3. The color of the nest label indicates its geographical nesting complex: light blue for Riaza, orange for Duratón, and purple for nests in other areas around. **(c)** Map showing the locations of Egyptian and cinereous vulture (*Aegypius monachus*) nests in the study area (Segovia, Spain). Point color corresponds to BFDV genetic clade (red for Clade 1, blue for Clade 2, and green for Clade 3), while point shape denotes species: circles for Egyptian vultures and triangles for cinereous vultures. Egyptian vulture nests that tested negative for BFDV are represented by smaller white dots. Background topography is based on a 30 m resolution digital elevation model from NASA Earth Observatory (http://earthobservatory.nasa.gov/). The regional boundaries of Spain were mapped using cartographic layers from the freely available database of the Instituto Geográfico Nacional (IGN; https://www.ign.es/web/ign/portal).

Phylogenetic analyses of BFDV sequences obtained in this study revealed three well supported major genetic clusters (BPP = 1.00), however, support values within clades were comparatively lower (Fig 2a). Clade 1 consisted of a monophyletic group (BPP = 1.00) comprising Egyptian vultures (refs: 24F, 1V9, 27L, 32M, 3C7, 32W) together with one cinereous vulture (93N). Within this clade, samples 32W and 93N formed a sister relationship (BPP = 1.00) to the remaining sequences, which were further divided into two subclades: one containing 1V9 and 24F (BPP = 0.91), and another containing 27L and the pair 3C7‑32M (BPP = 0.63). Clade 2 formed a distinct monophyletic group (refs: 32T, 9MC, 24N, 295, and 3UR), with 3UR as sister to the remainder of the clade (BPP = 0.66), and within this subclade, 295 as sister (BPP = 0.95) to a node comprising 24N, 9MC, and 32T (BPP = 1.00). Clade 3 was represented by a single sequence (246), forming a separate lineage sister to both previous clades (Fig 2a; BPP = 1.00). No sequences assigned to the same BFDV genetic clade were identified in individuals born in the same nest; instead, different lineages were identified in individuals born in the same nest in different years (Fig 2b). Spatially, the nests did not exhibit any apparent pattern of phylogeographic congruence among BFDV sequences (Fig 2c). Only in 2010 were samples from the same lineage detected in different nests of Egyptian vultures (Fig 2b).

**Fig 3:**
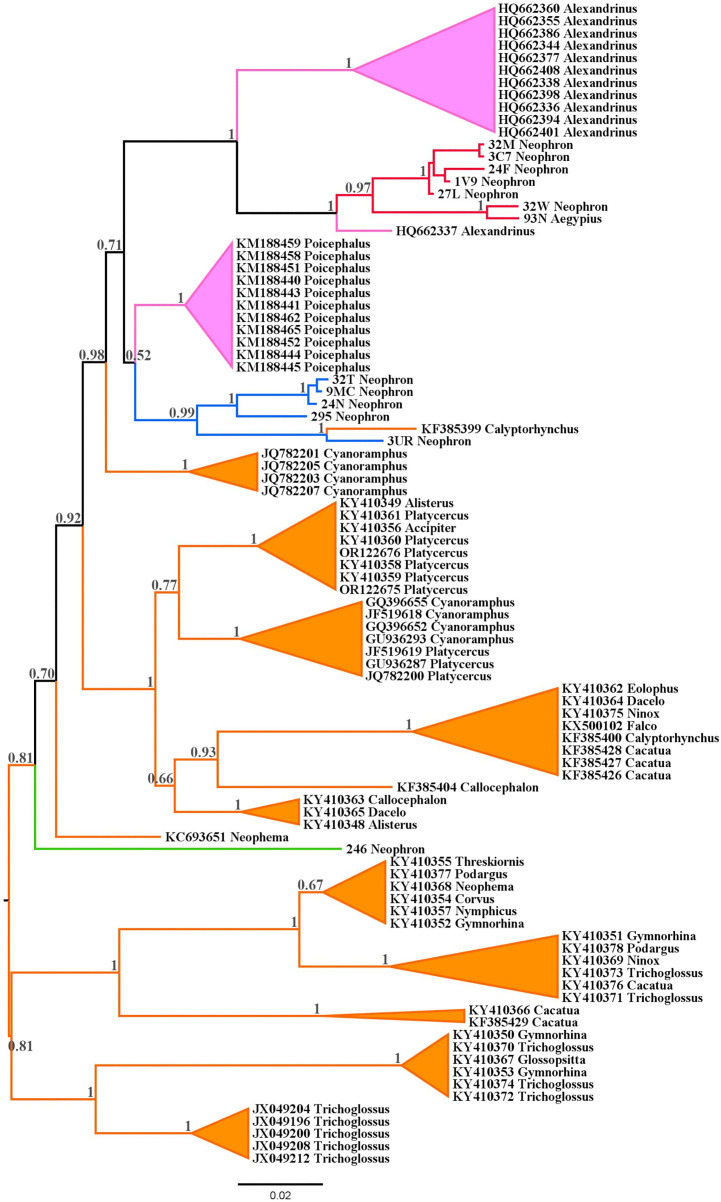
Phylogenetic relationships of BFDV strains from Old World vultures and other wild hosts. Phylogenetic tree of BFDV sequences obtained from wild hosts worldwide (sourced from GenBank) and from vulture nestlings (*Neophron percnopterus* and *Aegypius monachus*) sampled in Central Spain. Vulture BFDV-like sequences are color-coded according to their viral genetic clade: red for Clade 1, blue for Clade 2, and green for Clade 3. GenBank sequences of wild hosts are color-coded of their geographic origin as follows: orange for Oceania, and pink for Africa. GenBank accession numbers and host genera are indicated for each sequence. Numbers at branch nodes correspond to BPPs (Bayesian Posterior Probabilities).

Comparisons with published BFDV sequences from GenBank, derived from wild hosts spanning 29 species, 22 genera, 11 families, and 8 avian orders (S5 Table), revealed distinct phylogenetic structure across host taxa and geographic regions with relative well support (Fig 3). Vulture clade 1 grouped as sister to a *Alexandrinus eques* sequence from Mauritius (HQ662337; BPP = 1.00), and together they clustered with additional *A. eques* and feral *A. krameri* sequences from the same region (BPP = 1.00). Vulture clade 2, alongside a *Calyptorhynchus banksii* sequence from Australia (KF385399; BPP = 0.99), formed a sister group to a cluster of *P. robustus* sequences from South Africa (BPP = 0.52). Vulture clade 3 showed closer affinity to BFDV lineages previously described in Oceania with low support (BPP = 0.81). Notably, several sequences obtained from wild hosts in Australia and New Caledonia represented the most basal branches in the phylogeny (Fig 3; BPP = 0.81).

### Range overlap between Egyptian vultures and psittacines

Up to 28 psittacine species, both native and introduced as exotic species, somewhat overlap range with the Egyptian vulture (S6 Table). Notably, the native range of *A. krameri* overlapped with 19.7% of the vulture’s range. In the overwintering grounds of Iberian populations, Egyptian vulture range overlap with *P. senegalus* (44.9%), *A. krameri* (29.3%), and *P. fuscicollis* (4.6%) (S6 Table; S1 Fig). Among the psittacine species whose distributions overlap with that of the Egyptian vulture, 67.9% (19/28) have reported BFDV cases in the literature. However, only *A. krameri*, *P. robustus*, and *M. monachus* have documented positives in wild or introduced populations. (S6 Table). The overlapping ranges of the Egyptian vulture and both native and introduced parrot species, based on IUCN range data are shown in S1 Fig.

### Sequence similarities and genome integration of viral amplicons

Despite the phylogenetic pattern, BLASTn comparisons indicated that the closest sequence matches were primarily associated with worldwide captive psittacine hosts, although one sequence of a wild *A. eques* from Mauritius (HQ662337) shared close identities with vulture clade 1 individuals: 27L (96.6%), 1V9 (96.4%), 32M (95.6%), 3C7 (95.6%), and 24F (95.6%). The highest similarities observed (99.3%) were between BFDV detected in the Egyptian vulture 3UR and two captive hosts, specifically from a *Melopsittacus undulatus* from Slovakia (EU139454), and a *Psittacus erithacus* from Czech Republic (EU139461; S7 Table).

**Fig 4:**
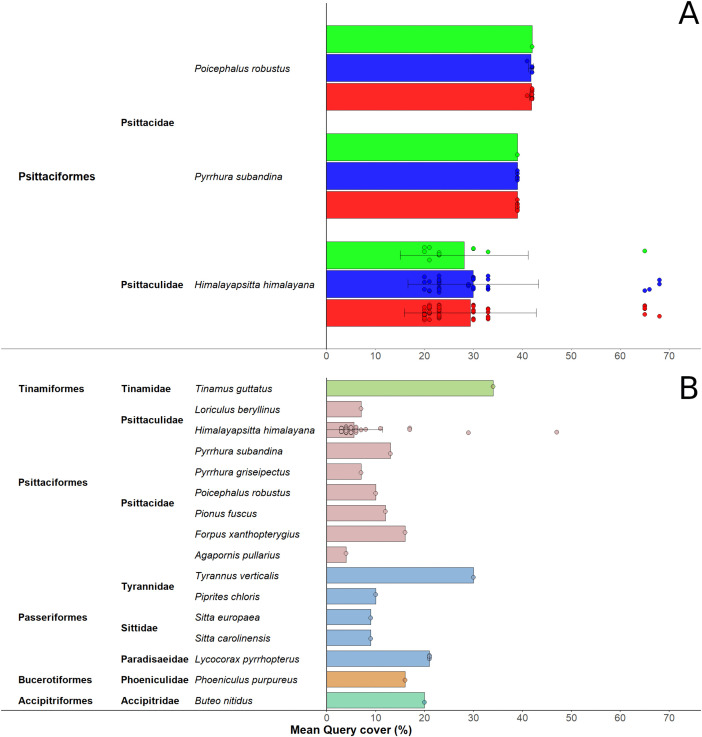
Mean query cover (%) of BFDV-like circoviral sequences detected across avian genomes. **(A)** Query cover of BFDV-like sequences identified in vulture genomes. Colors indicate the three major BFDV genetic clades found in vultures. **(B)** Query cover of a representative full-length BFDV genome (HM748919) detected across avian genomes. Species are grouped vertically by family (dotted horizontal lines) and by order (solid horizontal lines), with corresponding labels shown on the left. Colors denote taxonomic order. In both panels **(A–B)**, bars represent species-level mean query cover values, with horizontal error bars indicating ± standard deviation (SD).

BLASTg1 analysis of the BFDV sequences detected in vultures, revealed matches in the genomes of three avian species, suggesting the presence of putative endogenous circoviral elements related to the BFDV sequences detected in vultures (Fig 4a; S8 Table). Eleven of these hits were identified in *Himalayapsitta himalayana* genomes, with sequence similarities ranging from 20% to 68%. Additionally, single genome hits were observed for *P. robustus* (41–42% similarities) and *Pyrrhura subandina* (39% similarities) (Fig 4a; S8 Table). The BLASTg2 screening, which searched matches across the complete BFDV genome (HM748919) within avian genomes, revealed 16 worldwide taxa distributed among five orders and eight families, with sequence cover ranging from 47% to 3% (Fig 4b; S9 Table).

## Discussion

### Lack of evidence for an actively circulating virus

According to the hypothesis that the BFDV sequences detected in Old World vultures correspond to an actively circulating virus, several predictions should be supported by the results. Specifically, prevalence would be expected to correlate with ecological exposure to psittacines, infections should recur within nests or breeding territories across years, and phylogenetic structure should reveal clustering of related viral haplotypes within or between species, consistent with ongoing transmission [12,17,51]. However, our results fail to support any of these expectations.

Prevalence was low and spatially inconsistent across years and species, being higher in Egyptian vultures than in cinereous vultures, and undetected in griffon vultures. If an infectious BFDV were circulating, higher prevalence would be expected in griffon vultures than in cinereous vultures, since juveniles of the former species undertake regular migrations to the same African wintering areas as Egyptian vultures [28,29], where several psittacine species are present. By contrast, cinereous vultures perform only occasional and shorter-range movements into Africa [27]. Moreover, griffon vulture populations are much larger and the species is far more social than the other two vultures [32], which would favour a higher prevalence if a transmissible pathogen were involved. In any case, contact between these vulture species and psittacines in Africa is expected to be extremely limited due to the different habitats they occupy. Vultures mainly exploit open Sahelian savannas, whereas parrots are restricted to more southern, wooded regions of the Sudanian or Guinean regions [52].

No nest of Egyptian vulture was positive in consecutive years, and no pair of siblings tested positive simultaneously, even within the same breeding attempt. These results contradict the expectation of vertical or horizontal transmission within families or among neighbouring pairs. Environmental persistence of BFDV on contaminated surfaces or within reused nests [53] could theoretically enable repeated exposure across breeding seasons. However, the lack of repeated positives in reused nests or breeding territories argues against this mechanism. Egyptian vultures show strong nest-site fidelity [26,35], yet infection was not associated with specific nests or territories. Consequently, local persistence or reactivation of an exogenous pathogen appears unlikely. Moreover, no relationship emerged between BFDV detection and sex, brood size, hatching order, nest location, laying date, or body condition, implying that detection is not mediated by demographic, environmental, or physiological factors known to influence pathogen exposure or susceptibility [6,54–58].

At the genetic level, patterns of viral diversity also contradict an infectious origin. The presence of three highly divergent clades, with haplotypes from different years or nests interspersed within the same clades, indicates the absence of consistent phylogeographic or temporal structure. Even individuals born in the same nest but in different years carried unrelated haplotypes, while sequences from different species occasionally clustered together. This pattern is incompatible with continuous viral circulation within host populations, where replication and transmission typically generate geographically or host-specific clades [5,10,59].

The nearly complete absence of clinical symptoms also undermines the interpretation of an active infection. Only one Egyptian vulture nestling (individual 9MC) [26] exhibited PBFD-compatible clinical signs. It is crucial to note that this individual was the offspring of a brother-sister mating, representing an extreme case of inbreeding. Rather than an exogenous infection, the observed plumage malformations most likely stem from a genomic failure or the localized transcriptional expression of ECVs triggered by this compromised genetic background. Such alterations could also result from inbreeding-related physiological stress or metabolic disturbances linked to toxicity and associated disease [34]. The fact that these symptoms were exclusive to a single highly inbred individual, and did not spread to nest-mates or the wider population, strongly refutes the hypothesis of an active infectious outbreak and reinforces the conclusion that these sequences are stable, non-transmissible genomic elements. In fact, all other BFDV-positive and -negative individuals appeared clinically normal, as did all cinereous and griffon vultures. This mirrors findings in other non-psittacine birds, where BFDV sequences are frequently detected without associated pathology [7,8,14,18].

Finally, although Egyptian vultures overlap with several psittacine species in their African wintering grounds, the low prevalence and absence of clustering with African viral strains argue against exposure-driven infection. Migratory movements could, at most, provide sporadic opportunities for contact with contaminated environments, but not for sustained transmission cycles. Taken together, the epidemiological, genetic, and clinical evidence fails to support the existence of an actively circulating exogenous virus.

### Support for the endogenous (ECV) hypothesis

The alternative explanation that the BFDV-like sequences detected in vultures represent ancient endogenous ECV integrated into the host genome is supported by multiple independent lines of evidence.

First, the detected sequences displayed high conservation across individuals and years, coupled with the occurrence of divergent haplotypes even among siblings or within single nests. This pattern is characteristic of genomic insertions subject to Mendelian inheritance rather than horizontally transmitted viral infections [21,22]. The lack of consistent detection among related individuals and the absence of interannual persistence within nests suggest that PCR amplification captured stable, non-replicating DNA fragments rather than episomal viral genomes. The exclusive amplification of BFDV-like sequences using a single primer pair should not be interpreted as a technical artifact or primer-specific bias. On the contrary, the fact that these primers successfully amplified diverse haplotypes across multiple species and years, while consistently failing in non-vulture controls, argues for the high conservation and specificity of these elements within the vulture genome. This stability is a hallmark of ancient endogenization rather than the variability expected from an active circoviral infection.

Second, the closest BLASTg1 matches for vulture-derived amplicons corresponded to circoviral-like regions in the genomes of other bird species from Asia (*H. himalayana*), Africa (*P. robustus*), and South America (*P. subandina*), supporting the presence of homologous viral relics across distantly related taxa. Such shared viral fragments among unrelated species are typical of integrations inherited from a distant common ancestor, rather than resulting from recent viral jumps between hosts. These findings are consistent with previous reports showing that members of the Circoviridae family have integrated into the genomes of diverse vertebrates, including birds and mammals [22,60]. The detection of such elements across continents and host orders indicates a deep evolutionary origin, likely dating back millions of years.

Third, while some vulture sequences showed high similarity to BFDV variants from captive psittacines worldwide, this pattern most likely reflects database bias, since the overwhelming majority of available sequences derive from captive psittacines [17]. The observed affinities are thus better explained by shared ancestral fragments than by recent cross-species transmission.

The endogenous hypothesis also accounts for the absence of pathological manifestations. Endogenous viral elements are generally non-functional but can occasionally be transcribed under particular physiological or environmental conditions [21,61,62]. However, they do not produce complete viral genomes or infectious particles. Instead, they remain as molecular fossils within the host DNA, sometimes showing traces of former viral genes, which are no longer functional [22,60]. Moreover, the scattered and inconsistent detection of multiple divergent clades across individuals and years suggests that the amplified sequences correspond to distinct insertional variants of ancient viral origin. Similar findings have been reported for other circoviruses, such as pigeon circovirus (PiCV), where endogenous fragments have been found embedded within host genomes and occasionally co-amplified with exogenous viral DNA [22,63]. Hence, the heterogeneous genetic patterns found in vultures are more plausibly explained by multiple ancient integration events rather than contemporary infections.

### Evolutionary and ecological implications

An endogenous origin for the reported BFDV-like sequences has major implications for both viral ecology and wildlife disease surveillance. It reconciles the apparent contradiction between widespread BFDV-like detections across diverse avian taxa and the general absence of disease or transmission evidence. Rather than reflecting recent viral spread, these findings are consistent with the interpretation that they represent the genomic legacy of ancient circovirus–host interactions [22,60].

From an evolutionary perspective, the occurrence of BFDV-like ECVs in vultures, phylogenetically distant from psittacines, extends the known host range of circoviral integrations and supports a long history of viral endogenization across avian lineages. These elements may have been incorporated before the divergence of major bird orders, persisting as molecular fossils that occasionally are detected in PCR-based assays [60,23]. Their presence in the genomes of species occupying such different ecological niches underscores the ubiquity and evolutionary persistence of circoviruses.

These results also caution against interpreting PCR-based detection of BFDV as evidence of active infection in non-psittacine and psittacine hosts. Current diagnostic methods cannot easily discriminate between exogenous viral DNA and integrated genomic fragments, potentially leading to overestimation of infection prevalence [7,54,64]. Future approaches combining whole-genome sequencing, transcriptomic analyses, long-read sequencing, host–virus junction analyses‌‌, and flanking PCR could confirm the chromosomal integration of these sequences and definitively distinguish endogenous from exogenous signals.

Finally, our findings highlight the need to reinterpret the perceived risks associated with BFDV for wild bird populations. While continued surveillance remains important, especially for endangered taxa such as the Egyptian vulture, conservation strategies should not assume that detection equates to disease. Instead, emphasis should shift toward understanding how host genetics, environmental stressors, and evolutionary history modulate the sporadic expression of these ancient viral elements. This broader perspective will help prevent overestimating the pathogenic threat associated to BFDV sequences and refine conservation health assessments in the face of growing genomic complexity.

## Conclusions

Collectively, the evidence presented here, including low and inconsistent prevalence, absence of transmission or pathogenesis, high inter-lineage divergence, and cross-taxon genomic similarities, supports the endogenous circoviral element hypothesis. Accordingly, we propose that the BFDV-like sequences detected in Old World vultures most likely represent stable viral fragments integrated into the avian genome rather than actively circulating pathogens. Their occasional detection reflects the expression or amplification of ancient, inherited DNA remnants rather than ongoing viral replication. These findings challenge the conventional view of BFDV as a universally infectious psittacine virus and underscore the importance of integrating molecular virology, evolutionary genomics, and species-specific ecology to correctly interpret pathogen surveillance in wildlife. In particular, our findings suggest that many prior reports of avian circoviruses in non-psittacine hosts may have been confounded by the presence of ECVs. Finally, while direct molecular confirmation of integration sites was not the primary objective of this study, additional research is needed on junction-site sequencing to support our primary conclusions. Future research utilizing flanking PCR or long-read sequencing could provide additional molecular detail to further characterize these host-virus junctions.

## Supporting information

S1 Table Primers used to detect avian circovirus strains. Annealing temperature (Ta) used for this work and bird orders with birds BFDV positive previously reported are shown.
(PDF)

S2 Table Prevalence of BFDV in Egyptian vultures. This table presents the distribution of BFDV positive samples among tested Egyptian vultures sampled between 2004 and 2020. It details the number of BFDV-positive and negative individuals, and the associated prevalence (%) across different factors, including sex, brood size, nestling order, and geographical nest complex, and continuous variables as body condition and laying date. GLMM model averaging (ΔAICc < 2) p-values are shown.
(PDF)

S3 Table Summary of metadata for BFDV sequences detected in Old World vulture samples, comprising host species, individual ID codes, and associated GenBank accession numbers. (PDF)

S4 Table Genetic disparity matrix of the generated sequences. The number of nucleotide substitutions is shown below the diagonal, while the dissimilarity rate (%) based on sequence length is displayed above the diagonal. The matrix compares BFDV sequences from Egyptian vultures (*Neophron percnopterus*; Np) and cinereous vultures (*Aegypius monachus*; Am) hosts.
(PDF)

S5 Table BFDV sequences identified in wild birds retrieved from GenBank that were included in the phylogenetic framework for this study. The table provides GenBank accession numbers, taxonomic classification (order, family, and species), and country of origin for each sample.
(PDF)

S6 Table Percentage of range overlap between parrot species and the Egyptian vulture (*Neophron percnopterus*). This table presents the parrot species overlapping with Egyptian vulture range. The type of species area (introduced or native) and the continent where it is distributed (Africa -Af-; Asia -As-; Caribbean region -Ca-; Europe -Eu-; North America -NAm-; Oceania -Oc-), the percentage overlap within the entire range of the Egyptian vulture (as defined by the IUCN [37]), and the wintering range of parrot species in the Iberian Peninsula [29]. The presence of BFDV in each species is indicated as follows: (+) for confirmed cases, (–) for negative results, and (*) for positive cases recorded only in captive individuals.
(PDF)

S7 Table BLASTn search results for viral sequences obtained from vultures, filtered for ≥95% sequence identity to GenBank accession number. The table lists the GenBank accession numbers and percent identity of each match to the corresponding vulture sequence.
(PDF)

S8 Table Summary of BLASTg1 hits (≥ 20% query coverage) between vulture-derived BFDV sequences and whole-genome shotgun (WGS) contigs from avian species, showing potential endogenous viral elements (EVEs) integrations. (PDF)

S9 Table Summary of BLASTg2 hits (% query coverage) between the whole BFDV genome and whole-genome shotgun (WGS) contigs from avian species, showing potential endogenous viral elements (EVEs) integrations. (PDF)

S1 Fig Distribution map of the native range of the Egyptian vulture (*Neophron percnopterus*) and the distribution of parrot species. (a) Detailed map of the wintering grounds of Egyptian Vultures from the Iberian Peninsula [29], showing areas of overlap with the ranges of parrot species (*Alexandrinus krameri*: pink; *Poicephalus fuscicollis*: green; *Poicephalus senegalus*: blue). The map includes a zoomed-in view of Segovia (Spain), the study area, and the inferred migration routes of vulture juveniles. (b) General map of the Egyptian Vulture’s ranges (native breeding: dark green; native non-breeding; dark blue; passage; light blue; and native resident: light green) alongside the native (red) and introduced (orange) ranges of parrot species. Geographic ranges were derived from IUCN [37] polygons, created using QGIS (www.qgis.org). Country borders were obtained from Natural Earth (https://www.naturalearthdata.com/).
(TIF)
