# Supplementary material for: Not an infection: Endogenous circoviral elements underlie BFDV detections in Old World vultures
Source: PLoS One. 2026 Jun 15;21(6):e0351507. doi: 10.1371/journal.pone.0351507 (PMC13268160; doi:10.1371/journal.pone.0351507)
Supplement: S1 Table — Annealing temperature (Ta) used for this work and bird orders with birds BFDV positive previously reported are shown. (PDF) [file pone.0351507.s001.pdf]

S1 Table. Primers used to detect avian circovirus strains. Annealing temperature (Ta) used for this work and bird orders with birds BFDV positive previously reported are shown.

**Primer sequences (5′–3′)**	**Circovirus strains**	**Ta (ºC)**	**Reference**
Forward 5'-TTCACCCTTAAYAAYCCT-3'<br>Reverse 5'-CCRTSATATCCATCCCACCA-3'	Passeriformes,<br>Columbiformes<br>Anseriformes	52	[65,66]
Forward 5'-GCATAAGGTGCCCGTGAAAGG-3'<br>Reverse 5'-ATTCGCGGTCGCTCCGCT-3'	Columbiformes	60	[63]
Forward 5'-GGGTCCTCCTTGTAGTGGGATC-3'<br>Reverse 5'-CAGACGCCGTTTCACAACCAATAG-3'	Psittaciformes,<br>Passeriformes,<br>Anseriformes,<br>Caprimulgiformes,<br>Coraciiformes,<br>Strigiformes,<br>Pelecaniformes<br>Accipitriformes	58	[14]
Forward 5'-TTAACAACCCTACAGACGGCGA-3'<br>Reverse 5'-GGCGGAGCATCTCGCAATAAG-3'	Psittaciformes	58	[52]
