# Supplementary material for: Not an infection: Endogenous circoviral elements underlie BFDV detections in Old World vultures
Source: PLoS One. 2026 Jun 15;21(6):e0351507. doi: 10.1371/journal.pone.0351507 (PMC13268160; doi:10.1371/journal.pone.0351507)
Supplement: S2 Table — This table presents the distribution of BFDV positive samples among tested Egyptian vultures sampled between 2004 and 2020. It details the number of BFDV-positive and negative individuals, and the associated prevalence (%) across different factors, including sex, brood size, nestling order, and geographical nest complex, and continuous variables as body condition and laying date. GLMM model averaging (ΔAICc < 2) p-values are shown. (PDF) [file pone.0351507.s002.pdf]

S2 Table. Prevalence of BFDV in Egyptian vultures. This table presents the distribution of BFDV positive samples among tested Egyptian vultures sampled between 2004 and 2020. It details the number of BFDV-positive and negative individuals, and the associated prevalence (%) across different factors, including sex, brood size, nestling order, and geographical nest complex, and continuous variables as body condition and laying date. GLMM model averaging (ΔAICc < 2) p-values are shown.

			GLMM		
**Variable**	**Factor**	**BFDV test positive/negative (%)**	**Estimate**	**SE**	**p-value**
Sex	Females	6/76 (7.3)			
	Males	6/75 (7.4)	0.02	0.62	0.97
Brood size	1	6/58 (9.4)			
	2	6/92 (6.1)	-0.47	0.61	0.45
Hatching order	Single	6/58 (9.4)			
	First	4/46 (8.0)	-0.17	0.69	0.80
	Second	2/46 (4.2)	-0.87	0.85	0.31
Complex	Duratón	2/57 (3.4)			
	Riaza	7/57 (10.9)	1.16	0.83	0.16
	Other areas	2/37 (5.1)	0.78	0.96	0.42
Laying date		Mean (+): 329.8<br>Mean (-): 329.5	0.001	0.03	0.98
Body condition		Mean weight (+): 1,884.2<br>Mean weight (-): 1,832.0<br>Mean tarsus (+): 86.8<br>Mean tarsus (-): 86.2	3.06	6.15	0.62
