# Supplementary material for: Not an infection: Endogenous circoviral elements underlie BFDV detections in Old World vultures
Source: PLoS One. 2026 Jun 15;21(6):e0351507. doi: 10.1371/journal.pone.0351507 (PMC13268160; doi:10.1371/journal.pone.0351507)
Supplement: S3 Table — (PDF) [file pone.0351507.s003.pdf]

S3 Table. Summary of metadata for BFDV sequences detected in Old World vulture samples, comprising host species, individual ID codes, and associated GenBank accession numbers.

Species	ID code	GenBank Accession number
Neophron percnopterus	9MC	PX597144
Neophron percnopterus	32T	PX597145
Neophron percnopterus	32W	PX597146
Neophron percnopterus	3UR	PX597147
Neophron percnopterus	32M	PX597148
Neophron percnopterus	24N	PX597149
Neophron percnopterus	246	PX597150
Neophron percnopterus	27L	PX597151
Neophron percnopterus	24F	PX597152
Neophron percnopterus	1V9	PX597153
Neophron percnopterus	295	PX597154
Neophron percnopterus	3C7	PX597155
Aegypius monachus	93N	PX597156
