# Supplementary material for: Not an infection: Endogenous circoviral elements underlie BFDV detections in Old World vultures
Source: PLoS One. 2026 Jun 15;21(6):e0351507. doi: 10.1371/journal.pone.0351507 (PMC13268160; doi:10.1371/journal.pone.0351507)
Supplement: S4 Table — The number of nucleotide substitutions is shown below the diagonal, while the dissimilarity rate (%) based on sequence length is displayed above the diagonal. The matrix compares BFDV sequences from Egyptian vultures (Neophron percnopterus; Np) and cinereous vultures (Aegypius monachus; Am) hosts. (PDF) [file pone.0351507.s004.pdf]

S4 Table. Genetic disparity matrix of the generated sequences. The number of nucleotide substitutions is shown below the diagonal, while the dissimilarity rate (%) based on sequence length is displayed above the diagonal. The matrix compares BFDV sequences from Egyptian vultures (*Neophron percnopterus*; Np) and cinereous vultures (*Aegypius monachus*; Am) hosts.

	246_ Np	32W_ Np	32M_ Np	3C7_ Np	24F_ Np	27L_ Np	1V9_ Np	3UR_ Np	295_ Np	24N_ Np	32T_ Np	9MC_ Np	93N_ Am
246_Np	-	13.2	13.6	13.6	13.6	12.6	12.8	11.7	11.1	11.5	11.5	11.7	13.2
32W_Np	65	-	6.5	6.5	6.3	5.3	5.5	12.1	10.5	10.9	10.9	11.1	1.6
32M_Np	67	32	-	0.0	2.4	1.2	1.4	12.1	9.7	10.5	10.5	10.7	6.5
3C7_Np	67	32	0	-	2.4	1.2	1.4	12.1	9.7	10.5	10.5	10.7	6.5
24F_Np	67	31	12	12	-	1.2	1.0	11.5	9.5	9.7	9.7	9.9	5.9
27L_Np	62	26	6	6	6	-	0.2	11.3	8.7	9.3	9.3	9.5	5.3
1V9_Np	63	27	7	7	5	1	-	11.5	8.9	9.5	9.5	9.7	5.5
3UR_Np	58	60	60	60	57	56	57	-	7.3	7.1	7.5	7.3	12.8
295_Np	55	52	48	48	47	43	44	36	-	3.8	3.8	3.6	10.9
24N_Np	57	54	52	52	48	46	47	35	19	-	0.4	0.2	11.3
32T_Np	57	54	52	52	48	46	47	37	19	2	-	0.2	11.3
9MC_Np	58	55	53	53	49	47	48	36	18	1	1	-	11.5
93N_Am	65	8	32	32	29	26	27	63	54	56	56	57	-
