# Supplementary material for: Not an infection: Endogenous circoviral elements underlie BFDV detections in Old World vultures
Source: PLoS One. 2026 Jun 15;21(6):e0351507. doi: 10.1371/journal.pone.0351507 (PMC13268160; doi:10.1371/journal.pone.0351507)
Supplement: S5 Table — The table provides GenBank accession numbers, taxonomic classification (order, family, and species), and country of origin for each sample. (PDF) [file pone.0351507.s005.pdf]

S5 Table. BFDV sequences identified in wild birds retrieved from GenBank that were included in the phylogenetic framework for this study. The table provides GenBank accession numbers, taxonomic classification (order, family, and species), and country of origin for each sample.

GenBank Accession number	Order	Family	Species	Country
HQ662355	Psittaciformes	Psittaculidae	*Alexandrinus eques*	Mauritius
HQ662337	Psittaciformes	Psittaculidae	*Alexandrinus eques*	Mauritius
HQ662360	Psittaciformes	Psittaculidae	*Alexandrinus eques*	Mauritius
HQ662344	Psittaciformes	Psittaculidae	*Alexandrinus eques*	Mauritius
HQ662386	Psittaciformes	Psittaculidae	*Alexandrinus krameri*	Mauritius
HQ662377	Psittaciformes	Psittaculidae	*Alexandrinus eques*	Mauritius
HQ662398	Psittaciformes	Psittaculidae	*Alexandrinus krameri*	Mauritius
HQ662338	Psittaciformes	Psittaculidae	*Alexandrinus eques*	Mauritius
HQ662408	Psittaciformes	Psittaculidae	*Alexandrinus krameri*	Mauritius
HQ662401	Psittaciformes	Psittaculidae	*Alexandrinus krameri*	Mauritius
HQ662336	Psittaciformes	Psittaculidae	*Alexandrinus eques*	Mauritius
HQ662394	Psittaciformes	Psittaculidae	*Alexandrinus krameri*	Mauritius
KM188458	Psittaciformes	Psittacidae	*Poicephalus robustus*	South Africa
KM188440	Psittaciformes	Psittacidae	*Poicephalus robustus*	South Africa
KM188441	Psittaciformes	Psittacidae	*Poicephalus robustus*	South Africa
KM188445	Psittaciformes	Psittacidae	*Poicephalus robustus*	South Africa
KM188454	Psittaciformes	Psittacidae	*Poicephalus robustus*	South Africa
KM188455	Psittaciformes	Psittacidae	*Poicephalus robustus*	South Africa
KM188456	Psittaciformes	Psittacidae	*Poicephalus robustus*	South Africa
KY410356	Accipitriformes	Accipitridae	*Accipiter fasciatus*	Australia
KY410348	Psittaciformes	Psittaculidae	*Alisterus scapularis*	Australia
KY410349	Psittaciformes	Psittaculidae	*Alisterus scapularis*	Australia
KY410350	Passeriformes	Artamidae	*Gymnorhina tibicen*	Australia
KY410351	Passeriformes	Artamidae	*Gymnorhina tibicen*	Australia
KY410352	Passeriformes	Artamidae	*Gymnorhina tibicen*	Australia
KY410353	Passeriformes	Artamidae	*Gymnorhina tibicen*	Australia
KY410354	Passeriformes	Corvidae	*Corvus coronoides*	Australia
KY410355	Pelecaniformes	Threskiornithidae	*Threskiornis molucca*	Australia
KY410357	Psittaciformes	Cacatuidae	*Nymphicus hollandicus*	Australia
KY410358	Psittaciformes	Psittacidae	*Platycercus elegans*	Australia
KY410359	Psittaciformes	Psittacidae	*Platycercus elegans*	Australia
KY410360	Psittaciformes	Psittacidae	*Platycercus elegans*	Australia
KY410361	Psittaciformes	Psittacidae	*Platycercus eximius*	Australia
KY410362	Psittaciformes	Cacatuidae	*Eolophus roseicapilla*	Australia
KY410363	Psittaciformes	Cacatuidae	*Callocephalon fimbriatum*	Australia
KY410364	Coraciiformes	Alcedinidae	*Dacelo novaeguineae*	Australia
KY410365	Coraciiformes	Alcedinidae	*Dacelo novaeguineae*	Australia
KY410366	Psittaciformes	Cacatuidae	*Cacatua sanguinea*	Australia
KY410367	Psittaciformes	Psittaculidae	*Glossopsitta concinna*	Australia
KY410368	Psittaciformes	Psittaculidae	*Neophema chrysogaster*	Australia
KY410369	Strigiformes	Strigidae	*Ninox strenua*	Australia
KY410370	Psittaciformes	Psittaculidae	*Trichoglossus moluccanus*	Australia
KY410371	Psittaciformes	Psittaculidae	*Trichoglossus chlorolepidotus*	Australia
KY410372	Psittaciformes	Psittaculidae	*Trichoglossus chlorolepidotus*	Australia
KY410373	Psittaciformes	Psittaculidae	*Trichoglossus chlorolepidotus*	Australia
KY410374	Psittaciformes	Psittaculidae	*Trichoglossus chlorolepidotus*	Australia
KY410375	Strigiformes	Strigidae	*Ninox boobook*	Australia
KY410376	Psittaciformes	Cacatuidae	*Cacatua galerita*	Australia
KY410377	Caprimulgiformes	Podargidae	*Podargus strigoides*	Australia
KY410378	Caprimulgiformes	Podargidae	*Podargus strigoides*	Australia
JF519618	Psittaciformes	Psittaculidae	*Cyanoramphus novaezelandiae*	New Zealand
GQ396652	Psittaciformes	Psittaculidae	*Cyanoramphus novaezelandiae*	New Zealand
GQ396655	Psittaciformes	Psittaculidae	*Cyanoramphus novaezelandiae*	New Zealand

GU936287	Psittaciformes	Psittacidae	*Platycercus eximius*	New Zealand
GU936293	Psittaciformes	Psittaculidae	*Cyanoramphus novaezelandiae*	New Zealand
JQ782201	Psittaciformes	Psittaculidae	*Cyanoramphus auriceps*	New Zealand
JQ782203	Psittaciformes	Psittaculidae	*Cyanoramphus auriceps*	New Zealand
JQ782205	Psittaciformes	Psittaculidae	*Cyanoramphus auriceps*	New Zealand
JQ782207	Psittaciformes	Psittaculidae	*Cyanoramphus auriceps*	New Zealand
JF519619	Psittaciformes	Psittacidae	*Platycercus eximius*	New Zealand
JQ782200	Psittaciformes	Psittacidae	*Platycercus eximius*	New Zealand
KX500102	Falconiformes	Falconidae	*Falco peregrinus*	Australia
OR122675	Psittaciformes	Psittacidae	*Platycercus elegans*	Australia
OR122676	Psittaciformes	Psittacidae	*Platycercus elegans*	Australia
KC693651	Psittaciformes	Psittaculidae	*Neophema chrysogaster*	Australia
KF385399	Psittaciformes	Cacatuidae	*Calyptorhynchus banksii*	Australia
KF385429	Psittaciformes	Cacatuidae	*Cacatua tenuirostris*	Australia
KF385404	Psittaciformes	Cacatuidae	*Callocephalon fimbriatum*	Australia
KF385400	Psittaciformes	Cacatuidae	*Calyptorhynchus banksii*	Australia
KF385428	Psittaciformes	Cacatuidae	*Cacatua tenuirostris*	Australia
KF385426	Psittaciformes	Cacatuidae	*Cacatua tenuirostris*	Australia
KF385427	Psittaciformes	Cacatuidae	*Cacatua tenuirostris*	Australia
JX049196	Psittaciformes	Psittaculidae	*Trichoglossus haematodus deplanchii*	New Caledonia
JX049200	Psittaciformes	Psittaculidae	*Trichoglossus haematodus deplanchii*	New Caledonia
JX049204	Psittaciformes	Psittaculidae	*Trichoglossus haematodus deplanchii*	New Caledonia
JX049208	Psittaciformes	Psittaculidae	*Trichoglossus haematodus deplanchii*	New Caledonia
JX049212	Psittaciformes	Psittaculidae	*Trichoglossus haematodus deplanchii*	New Caledonia
