# Supplementary material for: Not an infection: Endogenous circoviral elements underlie BFDV detections in Old World vultures
Source: PLoS One. 2026 Jun 15;21(6):e0351507. doi: 10.1371/journal.pone.0351507 (PMC13268160; doi:10.1371/journal.pone.0351507)
Supplement: S6 Table — This table presents the parrot species overlapping with Egyptian vulture range. The type of species area (introduced or native) and the continent where it is distributed (Africa -Af-; Asia -As-; Caribbean region -Ca-; Europe -Eu-; North America -NAm-; Oceania -Oc-), the percentage overlap within the entire range of the Egyptian vulture (as defined by the IUCN [37]), and the wintering range of parrot species in the Iberian Peninsula [29]. The presence of BFDV in each species is indicated as follows: (+) for confirmed cases, (–) for negative results, and (*) for positive cases recorded only in captive individuals. (PDF) [file pone.0351507.s006.pdf]

S6 Table. Percentage of range overlap between parrot species and the Egyptian vulture (*Neophron percnopterus*). This table presents the parrot species overlapping with Egyptian vulture range. The type of species area (introduced or native) and the continent where it is distributed (Africa -Af-; Asia -As-; Caribbean region -Ca-; Europe -Eu-; North America -NAm-; Oceania -Oc-), the percentage overlap within the entire range of the Egyptian vulture (as defined by the IUCN [38]), and the wintering range of parrot species in the Iberian Peninsula [30]. The presence of BFDV in each species is indicated as follows: (+) for confirmed cases, (–) for negative results, and (*) for positive cases recorded only in captive individuals.

**Parrot species**	**Area (region)**	**Overall range (%)**	**Wintering range (%)**	**BFDV recorded (+ / – / *)**
Alexandrinus krameri	Native (Af, As)	**19.7**	**29.3**	*
	Introduced (Af, As, Eu, NAm, Oc)	1.0		+
Himalayapsitta cyanocephala	Native (As)	**8.5**		
Poicephalus meyeri	Native (Af)	**7.1**		*
Palaeornis eupatria	Native (As)	**5.9**		*
	Introduced (As)	0.03		
Poicephalus senegalus	Native (Af)	**5.3**	**44.9**	*
Poicephalus rufiventris	Native (Af)	**4.0**		*
Agapornis pullarius	Native (Af)	1.7		*
Agapornis taranta	Native (Af)	1.2		
Poicephalus fuscicollis	Native (Af)	1.2	**4.6**	
Loriculus vernalis	Native (As)	0.8		*
Myiopsitta monachus	Introduced (Eu, NAm)	0.8		+
Poicephalus rueppellii	Native (Af)	0.7		*
Poicephalus flavifrons	Native (Af)	0.6		
Agapornis roseicollis	Native (Af)	0.6		*
	Introduced (Af, NAm)	0.001		
Psittacula alexandri	Native (As)	0.5		*
	Introduced (As)	0.002		
Himalayapsitta himalayana	Native (As)	0.5		
Poicephalus gulielmi	Native (Af)	0.3		*
Nicopsitta columboides	Native (As)	0.3		

Poicephalus robustus	Native (Af)	0.3	+, *
Agapornis personatus	Native (Af)	0.3	*
	Introduced (Af)	0.03	
Agapornis fischeri	Native (Af)	0.3	*
	Introduced (Af, Ca)	0.1	
Agapornis lilianae	Native (Af)	0.1	*
Poicephalus cryptoxanthus	Native (Af)	0.1	*
Poicephalus crassus	Native (Af)	0.1	
Psittacus erithacus	Native (Af)	0.1	*
Himalayapsitta roseata	Native (As)	0.04	
Agapornis canus	Introduced (Af)	0.002	
Psittacus timneh	Native (Af)	0.0004	*
