# Supplementary material for: Not an infection: Endogenous circoviral elements underlie BFDV detections in Old World vultures
Source: PLoS One. 2026 Jun 15;21(6):e0351507. doi: 10.1371/journal.pone.0351507 (PMC13268160; doi:10.1371/journal.pone.0351507)
Supplement: S7 Table — The table lists the GenBank accession numbers and percent identity of each match to the corresponding vulture sequence. (PDF) [file pone.0351507.s007.pdf]

S7 Table. BLASTn search results for viral sequences obtained from vultures, filtered for ≥95% sequence identity to GenBank accession number. The table lists the GenBank accession numbers and percent identity of each match to the corresponding vulture sequence.

GenBank Accession Number	9MC	32T	24N	32W	3UR	32M	3C7	246	27l	24F	1V9	295	93N
HM748919	96.31	96.52	96.52	-	-	-	-	-	-	-	-	96.72	-
AB277732	96.17	96.17	96.17	-	-	-	-	-	-	-	-	96.62	-
MK803395	96.14	96.34	96.34	-	-	-	-	-	-	-	-	-	-
MH310937	96.13	96.13	96.13	-	-	-	-	-	-	-	-	96.13	-
AY450451	95.72	95.72	95.72	-	-	-	-	-	-	-	-	96.13	-
AY450443	95.32	95.32	95.32	-	-	-	-	-	-	-	-	96.13	-
MH279594	95.32	95.52	95.52	-	-	-	-	-	-	-	-	-	-
JX221039	95.3	95.50	95.50	-	-	-	-	-	-	-	-	-	-
JX221042	95.3	95.50	95.50	-	-	-	-	-	-	-	-	-	-
JX221038	95.09	95.30	95.30	-	-	-	-	-	-	-	-	-	-
HM748920	95.08	95.08	95.08	-	-	-	-	-	-	-	-	95.29	-
OR778790	-	-	-	97.36	-	-	-	-	95.12	-	-	-	97.57
AY518925	-	-	-	96.76	-	-	-	-	-	-	-	-	96.76
AY518908	-	-	-	96.36	-	-	-	-	-	-	-	-	96.36
AY518907	-	-	-	96.15	-	-	-	-	-	-	-	-	96.15
OR424522	-	-	-	95.55	-	-	-	-	-	-	-	-	95.14
OQ995063	-	-	-	95.55	-	-	-	-	-	-	-	-	95.14
OR424512	-	-	-	95.55	-	-	-	-	-	-	-	-	95.14
OQ995079	-	-	-	95.55	-	-	-	-	-	-	-	-	95.14
OQ995076	-	-	-	95.55	-	-	-	-	-	-	-	-	95.14
OR424514	-	-	-	95.55	-	-	-	-	-	-	-	-	95.14
OQ995091	-	-	-	95.55	-	-	-	-	-	-	-	-	95.14
OQ995081	-	-	-	95.55	-	95.76	95.76	-	96.97	96.36	96.77	-	95.75
OR424518	-	-	-	95.55	-	-	-	-	-	-	-	-	95.14
OR424509	-	-	-	95.55	-	-	-	-	-	-	-	-	95.14
OQ995069	-	-	-	95.55	-	-	-	-	-	-	-	-	95.14
OQ995090	-	-	-	95.55	-	-	-	-	-	-	-	-	95.14
OQ995070	-	-	-	95.55	-	-	-	-	-	-	-	-	95.14
OQ995086	-	-	-	95.55	-	-	-	-	-	-	-	-	95.14
OR424507	-	-	-	95.55	-	95.76	95.76	-	96.97	96.36	96.77	-	95.75
OQ995064	-	-	-	95.55	-	-	-	-	-	-	-	-	95.14
OQ995080	-	-	-	95.55	-	-	-	-	-	-	-	-	95.14
OR424508	-	-	-	95.55	-	-	-	-	-	-	-	-	95.14
OR424513	-	-	-	95.55	-	95.76	95.76	-	96.97	96.36	96.77	-	95.75
OQ995068	-	-	-	95.55	-	-	-	-	-	-	-	-	95.14
OQ995101	-	-	-	95.55	-	-	-	-	-	-	-	-	95.14
OR424510	-	-	-	95.55	-	-	-	-	-	-	-	-	95.14
OQ995058	-	-	-	95.55	-	-	-	-	-	-	-	-	95.14
OQ995083	-	-	-	95.55	-	-	-	-	-	-	-	-	95.14
OR424515	-	-	-	95.55	-	-	-	-	-	-	-	-	95.14
OR424525	-	-	-	95.55	-	-	-	-	-	-	-	-	95.14
OQ995098	-	-	-	95.55	-	-	-	-	-	-	-	-	95.14
OQ995097	-	-	-	95.55	-	-	-	-	-	-	-	-	95.14
OQ995085	-	-	-	95.55	-	-	-	-	-	-	-	-	95.14
OQ995084	-	-	-	95.55	-	-	-	-	-	-	-	-	95.14

OR424531	-	-	-	95.55	-	-	-	-	-	-	-	-	95.14
OR424528	-	-	-	95.55	-	-	-	-	-	-	-	-	95.14
OR424524	-	-	-	95.34	-	-	-	-	-	-	-	-	-
OQ995082	-	-	-	95.34	-	95.56	95.56	-	96.77	96.16	-	-	95.55
OQ995075	-	-	-	95.34	-	-	-	-	-	-	-	-	-
OQ995088	-	-	-	95.34	-	-	-	-	-	-	-	-	-
OR424529	-	-	-	95.34	-	95.56	95.56	-	96.77	96.16	96.57	-	95.55
OQ995078	-	-	-	95.34	-	-	-	-	-	-	-	-	-
OQ995065	-	-	-	95.34	-	-	-	-	-	-	-	-	-
OQ995061	-	-	-	95.34	-	-	-	-	-	-	-	-	-
OQ995071	-	-	-	95.34	-	-	-	-	-	-	-	-	-
OQ995059	-	-	-	95.34	-	-	-	-	-	-	-	-	-
OR424530	-	-	-	95.34	-	95.56	95.56	-	96.77	96.16	96.57	-	95.55
OR424511	-	-	-	95.34	-	-	-	-	-	-	-	-	-
OR424526	-	-	-	95.34	-	95.56	95.56	-	96.77	96.16	-	-	95.55
OQ995073	-	-	-	95.34	-	-	-	-	-	-	-	-	-
OQ995099	-	-	-	95.34	-	-	-	-	-	-	-	-	-
OR424519	-	-	-	95.34	-	-	-	-	-	-	-	-	-
OR424516	-	-	-	95.34	-	-	-	-	-	-	-	-	-
FJ685985	-	-	-	95.14	-	97.17	97.17	-	98.38	97.37	98.18	-	-
OQ995087	-	-	-	95.14	-	-	-	-	-	-	-	-	-
OR424527	-	-	-	95.14	-	95.35	95.35	-	96.57	95.96	96.36	-	95.34
OR424517	-	-	-	95.14	-	-	-	-	-	-	-	-	-
OQ995089	-	-	-	95.14	-	-	-	-	-	-	-	-	-
OQ995074	-	-	-	95.14	-	-	-	-	-	-	-	-	-
OR424523	-	-	-	95.14	-	-	-	-	-	-	-	-	-
OQ995077	-	-	-	95.14	-	-	-	-	-	-	-	-	-
EU139454	-	-	-	-	99.26	-	-	-	-	-	-	-	-
EU139461	-	-	-	-	99.26	-	-	-	-	-	-	-	-
JX221031	-	-	-	-	99.19	-	-	-	-	-	-	-	-
JX221030	-	-	-	-	99.19	-	-	-	-	-	-	-	-
JX221036	-	-	-	-	99.19	-	-	-	-	-	-	-	-
EU139440	-	-	-	-	99.01	-	-	-	-	-	-	-	-
JX221041	-	-	-	-	98.99	-	-	-	-	-	-	-	-
JX221037	-	-	-	-	98.99	-	-	-	-	-	-	-	-
JX049221	-	-	-	-	98.99	-	-	-	-	-	-	-	-
AY518927	-	-	-	-	98.99	-	-	-	-	-	-	-	-
KF723390	-	-	-	-	98.99	-	-	-	-	-	-	-	-
JX221035	-	-	-	-	98.99	-	-	-	-	-	-	-	-
JX049214	-	-	-	-	98.79	-	-	-	-	-	-	-	-
AY518904	-	-	-	-	98.79	-	-	-	-	-	-	-	-
KF723393	-	-	-	-	98.79	-	-	-	-	-	-	-	-
AY518928	-	-	-	-	98.79	-	-	-	-	-	-	-	-
JX049219	-	-	-	-	98.79	-	-	-	-	-	-	-	-
EU810207	-	-	-	-	98.79	-	-	-	-	-	-	-	-
EU139448	-	-	-	-	98.76	-	-	-	-	-	-	-	-
MH190788	-	-	-	-	98.58	-	-	-	-	-	-	-	-
MH180298	-	-	-	-	98.58	-	-	-	-	-	-	-	-
EU139451	-	-	-	-	98.51	-	-	-	-	-	-	-	-
JX049215	-	-	-	-	98.38	-	-	-	-	-	-	-	-
JX221033	-	-	-	-	98.37	-	-	-	-	-	-	-	-
JX049220	-	-	-	-	98.18	-	-	-	-	-	-	-	-
JX221032	-	-	-	-	98.17	-	-	-	-	-	-	-	-
AY521238	-	-	-	-	97.98	-	-	-	-	-	-	-	-
AY518899	-	-	-	-	97.77	-	-	-	-	-	-	-	-
AY518924	-	-	-	-	97.77	-	-	-	-	-	-	-	-
AY518920	-	-	-	-	97.57	-	-	-	-	-	-	-	-

AY518902	-	-	-	-	97.57	-	-	-	-	-	-	-	-
JX049216	-	-	-	-	97.57	-	-	-	-	-	-	-	-
AY518905	-	-	-	-	97.57	-	-	-	-	-	-	-	-
AY518915	-	-	-	-	97.37	-	-	-	-	-	-	-	-
JX049217	-	-	-	-	97.37	-	-	-	-	-	-	-	-
KF688553	-	-	-	-	97.17	-	-	-	-	-	-	-	-
JX049213	-	-	-	-	97.17	-	-	-	-	-	-	-	-
JX049218	-	-	-	-	96.96	-	-	-	-	-	-	-	-
AY521237	-	-	-	-	96.76	-	-	-	-	-	-	-	-
KF723398	-	-	-	-	96.76	-	-	-	-	-	-	-	-
KF723392	-	-	-	-	96.76	-	-	-	-	-	-	-	-
JX221006	-	-	-	-	96.76	-	-	-	-	-	-	-	-
JX221007	-	-	-	-	96.76	-	-	-	-	-	-	-	-
KF723397	-	-	-	-	96.76	-	-	-	-	-	-	-	-
JX221008	-	-	-	-	96.76	-	-	-	-	-	-	-	-
GQ329705	-	-	-	-	96.56	-	-	-	-	-	-	-	-
KF723399	-	-	-	-	96.56	-	-	-	-	-	-	-	-
KF723387	-	-	-	-	96.56	-	-	-	-	-	-	-	-
GU047347	-	-	-	-	96.56	-	-	-	-	-	-	-	-
KF723385	-	-	-	-	96.36	-	-	-	-	-	-	-	-
GQ120621	-	-	-	-	96.36	-	-	-	-	-	-	-	-
KF723400	-	-	-	-	96.36	-	-	-	-	-	-	-	-
KF723394	-	-	-	-	96.36	-	-	-	-	-	-	-	-
KF688552	-	-	-	-	96.15	-	-	-	-	-	-	-	-
KF723384	-	-	-	-	96.15	-	-	-	-	-	-	-	-
KF723402	-	-	-	-	96.15	-	-	-	-	-	-	-	-
KF723401	-	-	-	-	96.15	-	-	-	-	-	-	-	-
KF385399	-	-	-	-	96.15	-	-	-	-	-	-	-	-
KX449321	-	-	-	-	96.15	-	-	-	-	-	-	-	-
KF723386	-	-	-	-	96.15	-	-	-	-	-	-	-	-
OR778783	-	-	-	-	96.15	-	-	-	-	-	-	-	-
OR778782	-	-	-	-	95.95	-	-	-	-	-	-	-	-
MK803406	-	-	-	-	95.75	-	-	-	-	-	-	-	-
MK803407	-	-	-	-	95.55	-	-	-	-	-	-	-	-
MK803398	-	-	-	-	95.55	-	-	-	-	-	-	-	-
KF688549	-	-	-	-	95.55	-	-	-	-	-	-	-	-
KF688548	-	-	-	-	95.34	-	-	-	-	-	-	-	-
KM823541	-	-	-	-	95.14	-	-	-	-	-	-	-	-
MK803405	-	-	-	-	95.14	-	-	-	-	-	-	-	-
KP677574	-	-	-	-	-	96.15	96.15	-	97.36	96.35	97.16	-	-
HQ662337	-	-	-	-	-	95.56	95.56	-	96.57	95.56	96.36	-	-
OR778785	-	-	-	-	-	95.54	95.54	-	96.75	95.54	96.55	-	-
OR778787	-	-	-	-	-	95.30	95.30	-	96.52	95.30	96.32	-	-
KF723396	-	-	-	-	-	95.10	95.10	-	96.33	95.51	96.12	-	-
MK387074	-	-	-	-	-	-	-	98.38	-	-	-	-	-
MN720913	-	-	-	-	-	-	-	98.18	-	-	-	-	-
MK803399	-	-	-	-	-	-	-	97.18	-	-	-	-	-
MK387075	-	-	-	-	-	-	-	96.96	-	-	-	-	-
AY521234	-	-	-	-	-	-	-	-	95.56	-	95.35	-	-
AY521235	-	-	-	-	-	-	-	-	95.35	-	95.15	-	-
AY518919	-	-	-	-	-	-	-	-	95.35	-	95.15	-	-
OQ995104	-	-	-	-	-	-	-	-	95.16	-	-	-	-
AY518909	-	-	-	-	-	-	-	-	95.15	-	-	-	-
PV506316	-	-	-	-	-	-	-	-	-	-	-	-	95.11
