# Supplementary material for: Not an infection: Endogenous circoviral elements underlie BFDV detections in Old World vultures
Source: PLoS One. 2026 Jun 15;21(6):e0351507. doi: 10.1371/journal.pone.0351507 (PMC13268160; doi:10.1371/journal.pone.0351507)
Supplement: S8 Table — (PDF) [file pone.0351507.s008.pdf]

S8 Table. Summary of BLASTg1 hits (≥ 20 % query coverage) between vulture-derived BFDV sequences and whole-genome shotgun (WGS) contigs from avian species, showing potential endogenous viral elements (EVEs) integrations.

GenBank Accession number	Species	9MC_Np	32T_Np	24N_Np	32W_Np	3UR_Np	32M_Np	3C7_Np	246_Np	27l_Np	24F_Np	1V9_Np	295_Np	93N_Am
JAUHVH010119452	*Himalayapsitta himalayana*	68	68	68	65	-	65	65	65	65	65	65	66	65
JANDJS010088544	*Poicephalus robustus*	42	42	42	41	41	42	42	42	42	42	42	-	42
JARUHF010112971	*Pyrrhura subandina*	39	39	39	39	39	39	39	39	39	39	39	39	39
JAUHVH010082166	*Himalayapsitta himalayana*	33	33	33	33	33	33	33	33	33	33	33	33	33
JAUHVH010068634	*Himalayapsitta himalayana*	30	30	30	-	-	30	30	30	30	30	30	30	30
JAUHVH010012141	*Himalayapsitta himalayana*	30	30	30	23	29	30	30	30	30	30	30	30	29
JAUHVH010036668	*Himalayapsitta himalayana*	23	23	23	20	23	23	23	23	23	23	23	23	23
JAUHVH010002901	*Himalayapsitta himalayana*	23	23	23	23	23	23	23	23	23	23	23	23	23
JAUHVH010055235	*Himalayapsitta himalayana*	21	21	21	-	-	-	-	-	-	-	-	21	-
JAUHVH010090691	*Himalayapsitta himalayana*	-	-	-	20	20	20	20	20	-	-	-	20	-
JAUHVH010057888	*Himalayapsitta himalayana*	-	-	-	23	23	23	23	23	23	23	23	23	23
JAUHVH010066399	*Himalayapsitta himalayana*	-	-	-	-	-	21	21	21	-	-	21	23	-
JAUHVH010087980	*Himalayapsitta himalayana*	-	-	-	-	-	21	21	21	21	21	21	-	23
JAUHVH010045672	*Himalayapsitta himalayana*	-	-	-	-	-	20	20	20	20	20	20	-	20
