# Supplementary material for: Not an infection: Endogenous circoviral elements underlie BFDV detections in Old World vultures
Source: PLoS One. 2026 Jun 15;21(6):e0351507. doi: 10.1371/journal.pone.0351507 (PMC13268160; doi:10.1371/journal.pone.0351507)
Supplement: S9 Table — (PDF) [file pone.0351507.s009.pdf]

S9 Table. Summary of BLASTg2 hits (% query coverage) between the whole BFDV genome and whole-genome shotgun (WGS) contigs from avian species, showing potential endogenous viral elements (EVEs) integrations.

Species	Order	Family	Query cover (%)	Accession number
Himalayapsitta himalayana	Psittaciformes	Psittaculidae	47	JAUHVH010067744
Tinamus guttatus	Tinamiformes	Tinamidae	34	JMFW02027068
Tyrannus verticalis	Passeriformes	Tyrannidae	30	JAUCNF010001520
Himalayapsitta himalayana	Psittaciformes	Psittaculidae	29	JAUHVH010036668
Lycocorax pyrrhopterus	Passeriformes	Paradisaeidae	21	JACSVV010000009
Lycocorax pyrrhopterus	Passeriformes	Paradisaeidae	21	JACTOI010000337
Lycocorax pyrrhopterus	Passeriformes	Paradisaeidae	21	JACSVZ010000001
Lycocorax pyrrhopterus	Passeriformes	Paradisaeidae	21	JACSVR010000001
Buteo nitidus	Accipitriformes	Accipitridae	20	JANVCP010002506
Himalayapsitta himalayana	Psittaciformes	Psittaculidae	17	JAUHVH010119452
Himalayapsitta himalayana	Psittaciformes	Psittaculidae	17	JAUHVH010105937
Forpus xanthopterygius	Psittaciformes	Psittacidae	16	JASSWD010131657
Phoeniculus purpureus	Bucerotiformes	Phoeniculidae	16	JAOXOE010000343
Pyrrhura subandina	Psittaciformes	Psittacidae	13	JARUHF010112971
Pionus fuscus	Psittaciformes	Psittacidae	12	JARWLP010226903
Himalayapsitta himalayana	Psittaciformes	Psittaculidae	11	JAUHVH010072387
Poicephalus robustus	Psittaciformes	Psittacidae	10	JANDJS010088544
Piprites chloris	Passeriformes	Tyrannidae	10	VXAH01000762
Sitta carolinensis	Passeriformes	Sittidae	9	JAVUNC010000007
Sitta europaea	Passeriformes	Sittidae	9	VXBS01002389
Himalayapsitta himalayana	Psittaciformes	Psittaculidae	8	JAUHVH010082166
Himalayapsitta himalayana	Psittaciformes	Psittaculidae	7	JAUHVH010068634
Himalayapsitta himalayana	Psittaciformes	Psittaculidae	7	JAUHVH010012141
Loriculus beryllinus	Psittaciformes	Psittaculidae	7	JBCJKH010085419
Pyrrhura griseipectus	Psittaciformes	Psittacidae	7	JAAAKS020103574
Himalayapsitta himalayana	Psittaciformes	Psittaculidae	6	JAUHVH010057888
Himalayapsitta himalayana	Psittaciformes	Psittaculidae	6	JAUHVH010010302
Himalayapsitta himalayana	Psittaciformes	Psittaculidae	6	JAUHVH010049882
Himalayapsitta himalayana	Psittaciformes	Psittaculidae	6	JAUHVH010056402
Himalayapsitta himalayana	Psittaciformes	Psittaculidae	6	JAUHVH010055235
Himalayapsitta himalayana	Psittaciformes	Psittaculidae	6	JAUHVH010045672
Himalayapsitta himalayana	Psittaciformes	Psittaculidae	6	JAUHVH010002901
Himalayapsitta himalayana	Psittaciformes	Psittaculidae	6	JAUHVH010066399
Himalayapsitta himalayana	Psittaciformes	Psittaculidae	5	JAUHVH010050048
Himalayapsitta himalayana	Psittaciformes	Psittaculidae	5	JAUHVH010087980
Himalayapsitta himalayana	Psittaciformes	Psittaculidae	5	JAUHVH01008172
Himalayapsitta himalayana	Psittaciformes	Psittaculidae	5	JAUHVH010047751
Himalayapsitta himalayana	Psittaciformes	Psittaculidae	5	JAUHVH010090691
Himalayapsitta himalayana	Psittaciformes	Psittaculidae	5	JAUHVH010027994
Himalayapsitta himalayana	Psittaciformes	Psittaculidae	5	JAUHVH010085315
Himalayapsitta himalayana	Psittaciformes	Psittaculidae	5	JAUHVH010074089
Himalayapsitta himalayana	Psittaciformes	Psittaculidae	5	JAUHVH010078967
Himalayapsitta himalayana	Psittaciformes	Psittaculidae	5	JAUHVH010000948
Himalayapsitta himalayana	Psittaciformes	Psittaculidae	4	JAUHVH010091416
Himalayapsitta himalayana	Psittaciformes	Psittaculidae	4	JAUHVH010020943
Himalayapsitta himalayana	Psittaciformes	Psittaculidae	4	JAUHVH010118370
Himalayapsitta himalayana	Psittaciformes	Psittaculidae	4	JAUHVH010039970

Himalayapsitta himalayana	Psittaciformes	Psittaculidae	4	JAUHVH010029154
Himalayapsitta himalayana	Psittaciformes	Psittaculidae	4	JAUHVH010022428
Himalayapsitta himalayana	Psittaciformes	Psittaculidae	4	JAUHVH010070241
Himalayapsitta himalayana	Psittaciformes	Psittaculidae	4	JAUHVH010064761
Himalayapsitta himalayana	Psittaciformes	Psittaculidae	4	JAUHVH010056114
Himalayapsitta himalayana	Psittaciformes	Psittaculidae	4	JAUHVH010000730
Himalayapsitta himalayana	Psittaciformes	Psittaculidae	4	JAUHVH010031603
Himalayapsitta himalayana	Psittaciformes	Psittaculidae	4	JAUHVH010070067
Himalayapsitta himalayana	Psittaciformes	Psittaculidae	4	JAUHVH010044754
Agapornis pullarius	Psittaciformes	Psittacidae	4	JAVKLG010126073
Himalayapsitta himalayana	Psittaciformes	Psittaculidae	4	JAUHVH010093183
Himalayapsitta himalayana	Psittaciformes	Psittaculidae	4	JAUHVH010010084
Himalayapsitta himalayana	Psittaciformes	Psittaculidae	4	JAUHVH010020266
Himalayapsitta himalayana	Psittaciformes	Psittaculidae	4	JAUHVH010119604
Himalayapsitta himalayana	Psittaciformes	Psittaculidae	4	JAUHVH010026124
Himalayapsitta himalayana	Psittaciformes	Psittaculidae	4	JAUHVH010056850
Himalayapsitta himalayana	Psittaciformes	Psittaculidae	4	JAUHVH010007717
Himalayapsitta himalayana	Psittaciformes	Psittaculidae	4	JAUHVH010084706
Himalayapsitta himalayana	Psittaciformes	Psittaculidae	4	JAUHVH010035164
Himalayapsitta himalayana	Psittaciformes	Psittaculidae	4	JAUHVH010011148
Himalayapsitta himalayana	Psittaciformes	Psittaculidae	4	JAUHVH010049596
Himalayapsitta himalayana	Psittaciformes	Psittaculidae	4	JAUHVH010129685
Himalayapsitta himalayana	Psittaciformes	Psittaculidae	4	JAUHVH010084001
Himalayapsitta himalayana	Psittaciformes	Psittaculidae	4	JAUHVH010082928
Himalayapsitta himalayana	Psittaciformes	Psittaculidae	4	JAUHVH010078708
Himalayapsitta himalayana	Psittaciformes	Psittaculidae	4	JAUHVH010076564
Himalayapsitta himalayana	Psittaciformes	Psittaculidae	4	JAUHVH010016773
Himalayapsitta himalayana	Psittaciformes	Psittaculidae	4	JAUHVH010083191
Himalayapsitta himalayana	Psittaciformes	Psittaculidae	4	JAUHVH010078226
Himalayapsitta himalayana	Psittaciformes	Psittaculidae	4	JAUHVH010034766
Himalayapsitta himalayana	Psittaciformes	Psittaculidae	4	JAUHVH010133914
Himalayapsitta himalayana	Psittaciformes	Psittaculidae	4	JAUHVH010034462
Himalayapsitta himalayana	Psittaciformes	Psittaculidae	4	JAUHVH010033144
Himalayapsitta himalayana	Psittaciformes	Psittaculidae	4	JAUHVH010084291
Himalayapsitta himalayana	Psittaciformes	Psittaculidae	4	JAUHVH010063813
Himalayapsitta himalayana	Psittaciformes	Psittaculidae	4	JAUHVH010029723
Himalayapsitta himalayana	Psittaciformes	Psittaculidae	4	JAUHVH010028223
Himalayapsitta himalayana	Psittaciformes	Psittaculidae	4	JAUHVH010019288
Himalayapsitta himalayana	Psittaciformes	Psittaculidae	4	JAUHVH010017589
Himalayapsitta himalayana	Psittaciformes	Psittaculidae	4	JAUHVH010076527
Himalayapsitta himalayana	Psittaciformes	Psittaculidae	4	JAUHVH010047689
Himalayapsitta himalayana	Psittaciformes	Psittaculidae	4	JAUHVH010069531
Himalayapsitta himalayana	Psittaciformes	Psittaculidae	4	JAUHVH010027653
Himalayapsitta himalayana	Psittaciformes	Psittaculidae	4	JAUHVH010026552
Himalayapsitta himalayana	Psittaciformes	Psittaculidae	4	JAUHVH010068870
Himalayapsitta himalayana	Psittaciformes	Psittaculidae	4	JAUHVH010040556
Himalayapsitta himalayana	Psittaciformes	Psittaculidae	3	JAUHVH010145147
Himalayapsitta himalayana	Psittaciformes	Psittaculidae	3	JAUHVH010042479
Himalayapsitta himalayana	Psittaciformes	Psittaculidae	3	JAUHVH010091144
Himalayapsitta himalayana	Psittaciformes	Psittaculidae	3	JAUHVH010000924
Himalayapsitta himalayana	Psittaciformes	Psittaculidae	3	JAUHVH010058260
Himalayapsitta himalayana	Psittaciformes	Psittaculidae	3	JAUHVH010020730
Himalayapsitta himalayana	Psittaciformes	Psittaculidae	3	JAUHVH010122677
